# Internalized criticism and absence of care: separate pathways from childhood abuse and neglect to dual harm via self-compassion

**DOI:** 10.3389/fpsyt.2026.1762268

**Published:** 2026-02-10

**Authors:** Yanxia Mao, Zhongguang Xie, Liying Zhang, Wenchao Wang

**Affiliations:** 1 School of Education Science, Luoyang Normal University, Luoyang, China; 2 Beijing Key Laboratory of Applied Experimental Psychology, Faculty of Psychology, Beijing Normal University, Beijing, China

**Keywords:** child abuse, child neglect, cyber aggression, non-suicidal self-injury, self-compassion

## Abstract

**Background:**

Non-suicidal self-injury and cyber aggression are increasingly prevalent among college students. Childhood maltreatment is a known risk factor for both behaviors, yet few studies have examined their co-occurrence (dual harm) or the underlying mechanisms from a developmental psychopathology perspective. This study investigates the distinct effects of childhood abuse and neglect on NSSI and cyber aggression, with self-compassion as a potential mediator.

**Methods:**

A longitudinal design with a six-month interval was employed. A sample of 2,301 Chinese college students completed the Childhood Trauma Questionnaire-Short Form and the Self-Compassion Scale at baseline, followed by the Inventory of Statements about Self-Injury and the Adolescent Cyber Aggression Scale at follow-up. Structural equation modeling and bootstrapping were used to examine direct and mediated pathways.

**Results:**

Childhood abuse and neglect positively predicted both NSSI and cyber aggression. Negative self-compassion mediated the relationship between childhood abuse and both outcomes. For childhood neglect, positive self-compassion mediated its positive association with NSSI and cyber aggression, while negative self-compassion mediated a negative association.

**Conclusions:**

The findings highlight differential pathways through which childhood abuse and neglect influence later self- and other-directed harm. Positive self-compassion may serve as a protective factor, whereas negative self-compassion may exacerbate risk. Tailored interventions focusing on enhancing self-compassion could help mitigate the long-term effects of childhood maltreatment.

## 1 Introduction

The transition to adulthood, particularly the college years, represents a critical developmental period characterized by significant psychological, social, and academic challenges. During this phase of emerging adulthood (approximately ages 18-25), individuals navigate the complexities of identity formation, increased autonomy, and the establishment of independent life trajectories (1). While often idealized as a time of exploration and opportunity, this period is also marked by heightened vulnerability to mental health difficulties due to its inherent instability and the convergence of multiple stressors (2). Among the spectrum of psychopathological and behavioral concerns observed in college populations, non-suicidal self-injury (NSSI) and cyber aggression have emerged as two particularly prevalent and debilitating issues, drawing increasing attention from researchers and mental health practitioners worldwide.

NSSI, defined as the deliberate, self-inflicted damage of body tissue without suicidal intent and for purposes not socially sanctioned, manifests in behaviors such as cutting, burning, or hitting oneself (3). Far from being a benign coping mechanism, NSSI is a significant risk factor for subsequent suicidal ideation and attempts, with self-injuring individuals found to be at a markedly elevated risk for suicide (4). Epidemiological studies indicate troubling prevalence rates among college students, with meta-analytic estimates suggesting approximately 13-21% of undergraduates engage in NSSI at some point (5–7). Concurrently, the digital age has given rise to a novel form of interpersonal harm: cyber aggression. This behavior involves the intentional use of information and communication technologies to inflict harm on others, encompassing harassment, verbal attacks, social exclusion, and rumor-spreading online (8, 9). The pervasive nature of the internet, combined with features such as anonymity, reduced social feedback, and constant accessibility, has made cyber aggression a common experience, with studies reporting that over 60% of Chinese university students and 72% of students internationally have engaged in such behavior (10).

Traditionally, NSSI and aggression have been studied within separate clinical and research silos. NSSI is typically framed as an indicator of intrapersonal distress and a self-directed behavior often evoking clinical concern and caregiving responses (11, 12). In contrast, aggression, especially its cyber variant, is viewed as an interpersonal, externalizing behavior directed towards others, often eliciting social condemnation and punitive responses. However, a growing body of evidence challenges this dichotomy, suggesting that self-directed and other-directed harms frequently co-occur—a phenomenon termed “dual harm” (12). Empirical reviews indicate substantial overlap, with co-occurrence rates of self-harm and outward aggression ranging from 15% to over 70% across diverse samples (13). This co-occurrence suggests shared etiological pathways and common underlying vulnerabilities. From a psychodynamic perspective, both behaviors can be understood as manifestations of aggression, either internalized (as in NSSI) or externalized (as in cyber aggression) (14). This integrative view necessitates research that simultaneously examines both behavioral outcomes to uncover their shared and unique antecedents.

A robust and consistent predictor of both NSSI and aggression is a history of childhood maltreatment. Childhood maltreatment, encompassing experiences of abuse (physical, emotional, sexual) and neglect (physical, emotional), represents a severe violation of a child’s safety and developmental needs, with profound and enduring consequences for psychological well-being (15). It is a global public health crisis, with meta-analytic findings indicating high prevalence rates worldwide (16). The link between childhood trauma and later psychopathology is well-established, with maltreatment history serving as a potent risk factor for depression, anxiety, substance abuse, and importantly, both NSSI and aggressive behaviors in adolescence and adulthood (17–19). For instance, childhood emotional neglect has been strongly linked to NSSI (20), while experiences of abuse are significant predictors of later aggression, including its online forms (21).

Despite this established link, critical gaps persist in the literature. First, much of the prior research has operationalized childhood adversity using a cumulative risk approach, summing various adverse experiences into a single score (22). While highlighting the dose-response relationship between adversity and poor outcomes, this approach obscures the potentially distinct psychological impacts of different types of adversity. It implicitly assumes that diverse traumatic experiences operate through identical mechanisms, which is increasingly recognized as an oversimplification. To address this, McLaughlin and Sheridan (23) proposed the Dimensional Model of Adversity, which distinguishes between two core dimensions: threat (experiences involving harm or threat of harm, such as physical/emotional abuse) and deprivation (experiences involving the absence of expected environmental input and care, such as physical/emotional neglect). This model posits that threat and deprivation may differentially impact neurodevelopmental pathways, emotional learning, and ultimately, psychopathological outcomes. Emerging evidence supports this distinction; threat-related maltreatment (abuse) is more consistently linked to externalizing problems and hypervigilance to danger, while deprivation-related maltreatment (neglect) shows stronger associations with internalizing problems, cognitive deficits, and blunted emotional responsiveness (23–25). Therefore, a nuanced investigation separating the effects of childhood abuse (threat) and neglect (deprivation) on dual harm outcomes is not only theoretically warranted but essential for developing targeted interventions.

Second, the psychological mechanisms that transmit the risk from early maltreatment to later dual harm are not fully understood. Theoretical models, such as the Cognitive-Emotional Model of Dual Harm (14) and Yates’s (26) Developmental Psychopathology Model of NSSI, emphasize the role of maladaptive cognitive-emotional schemas formed in response to early trauma. These schemas, which include core beliefs about the self, others, and the world, shape an individual’s emotion regulation strategies. When faced with stress, individuals with negative schemas and poor emotion regulation are more likely to engage in dysregulated behaviors, such as NSSI or aggression, as a means of coping with overwhelming affect (27). Within this framework, self-compassion emerges as a critical emotion regulation construct worthy of detailed examination (28).

Self-compassion, rooted in Buddhist philosophy and operationalized by Neff (29), involves treating oneself with kindness, understanding, and a non-judgmental attitude during times of failure, inadequacy, or suffering. Crucially, contemporary research conceptualizes it not as a unidimensional trait but as comprising two related but distinct components: positive self-compassion (encompassing self-kindness, common humanity, and mindfulness) and negative self-compassion (encompassing self-judgment, isolation, and over-identification). Positive self-compassion acts as an adaptive, soothing emotion regulation strategy, helping individuals to acknowledge pain without avoidance or harsh self-criticism, thereby reducing the need for extreme behavioral outlets (30, 31). In contrast, negative self-compassion reflects a ruminative, self-critical, and isolated stance towards one’s suffering, which can amplify distress and impede effective coping (32).

There is strong evidence linking self-compassion to the outcomes of interest. Higher levels of positive self-compassion are associated with lower levels of NSSI (33, 34) and reduced aggression (35, 36). Conversely, higher levels of negative self-compassion are linked to increased risk for NSSI (37) and aggressive tendencies (38). Furthermore, childhood maltreatment is a known antecedent of low self-compassion. Abusive and neglectful caregiving environments undermine the development of a secure, comforting inner voice, often replacing it with an internalized critical one, thereby stifling positive self-compassion and fostering negative self-compassion (15, 39). However, scant research has investigated whether abuse and neglect exert differential effects on these two components of self-compassion. Following the Dimensional Model, one might hypothesize that threat (abuse) could more directly foster negative self-compassion (e.g., self-judgment aligned with external criticism), while deprivation (neglect), through the absence of nurturing, might more strongly inhibit the development of positive self-compassion (e.g., self-kindness).

Third, the existing literature on these relationships in college student populations, especially within Chinese cultural contexts, remains limited. Culturally, Chinese parenting practices have historically emphasized strict discipline and academic achievement, with physical punishment sometimes normalized as “jiajiao” (family education) rather than labeled as abuse (40). Similarly, emotional expression and needs may be undervalued, potentially increasing the prevalence and normative perception of emotional neglect. These cultural nuances may shape the perception, reporting, and psychological impact of childhood maltreatment. Furthermore, the collectivistic orientation of Chinese society, which prioritizes social harmony, may influence the expression of aggression, potentially diverting it from face-to-face confrontations to the more anonymous and disinhibited realm of cyberspace, where it aligns with the externalizing pathway of dual harm (14). Therefore, examining dual harm and its precursors in a Chinese university sample is critical for developing culturally informed models and interventions.

Finally, methodological limitations persist. Many studies in this area are cross-sectional, precluding causal inference and the examination of temporal processes. Longitudinal designs are necessary to establish predictive relationships and better test mediating pathways over time (41).

### 1.1 The present study

To address these significant gaps, this longitudinal study investigates the distinct influences of childhood abuse (threat) and childhood neglect (deprivation) on later NSSI and cyber aggression (dual harm) in a large sample of Chinese college students. Drawing on the Dimensional Model of Adversity and the Cognitive-Emotional Model of Dual Harm, we propose that self-compassion is a critical mechanism explaining these links. Crucially, we hypothesize that abuse and neglect will relate to the two dimensions of self-compassion in distinct ways, leading to divergent pathways to harm.

We propose the following specific hypotheses:

H1: Both childhood abuse and neglect at Time 1 (T1) will positively predict levels of NSSI and cyber aggression at Time 2 (T2).
H2: Self-compassion will mediate these relationships, but the pathways will differ by maltreatment type:
H2a: Childhood abuse will positively predict NSSI and cyber aggression through its positive association with negative self-compassion.
H2b: Childhood neglect will positively predict NSSI and cyber aggression through its negative association with positive self-compassion.

By testing these hypotheses, this study aims to advance theoretical understanding of the developmental pathways to dual harm, highlight the importance of distinguishing between threat and deprivation, and identify specific self-compassion components that could be targeted in preventive and therapeutic interventions for university students with histories of childhood trauma.

## 2 Methods

### 2.1 Participants and procedure

The present study employed a two-wave longitudinal design with a six-month interval between assessments. Data collection was conducted at a large public university located in Central China. Initial recruitment employed a convenience cluster sampling method, where entire class cohorts from the freshman and sophomore years were invited to participate to ensure a diverse representation of students across majors. At Time 1 (T1, October 2022), a total of 3,720 undergraduate students provided informed consent and completed the initial battery of questionnaires. The study procedures, including the consent forms, were reviewed and approved by the Institutional Review Board of the Faculty of Psychology at Beijing Normal University (Approval No. 12220085). All participants were informed of the voluntary nature of their participation, their right to withdraw at any time without penalty, and the confidentiality of their responses.

At T1, we administered the Childhood Trauma Questionnaire-Short Form (CTQ-SF) as a screening tool. Participants who scored at or above the established “low to moderate” threshold for at least one subtype of maltreatment (emotional abuse, physical abuse, emotional neglect, or physical neglect) were retained for the longitudinal follow-up (42). This screening criterion was implemented to enrich our sample with individuals who had experienced at least some level of maltreatment, thereby increasing the variance on our key predictor variables and enhancing our power to detect relationships with outcomes. However, it is important to note that this strategy means our findings are most directly generalizable to college student populations with a non-trivial history of childhood adversity, rather than to the full spectrum of students with zero to severe maltreatment. At Time 2 (T2, April 2023), six months later, all eligible participants from T1 were re-contacted via class coordinators to complete the follow-up survey. Attrition between waves was primarily due to typical academic transitions (e.g., graduation of some seniors, students being absent during survey administration). We conducted attrition analyses comparing the baseline scores on all T1 study variables (childhood abuse, neglect, self-compassion) and demographic factors (gender, age) between those who participated at T2 and those who did not. No significant differences were found on any of these variables, suggesting that the follow-up sample was representative of the initial screened cohort and that attrition was not systematically related to the study’s primary variables.

The final longitudinal sample for analysis consisted of 2,301 students (1,127 females, 53.3%; 1,174 males, 46.7%) who met the maltreatment screening criterion and provided complete data at both time points. Participants ranged in age from 18 to 23 years (*M* = 18.94, *SD* = 1.94).

### 2.2 Measures

#### 2.2.1 Childhood maltreatment (T1)

Childhood maltreatment was assessed using the CTQ-SF (43). The CTQ-SF is a 28-item retrospective self-report inventory with excellent psychometric properties, designed to screen for histories of abuse and neglect before age 16. It yields five clinical subscales: Emotional Abuse (EA), Physical Abuse (PA), Sexual Abuse (SA), Emotional Neglect (EN), and Physical Neglect (PN). Each subscale comprises 5 items rated on a 5-point Likert scale from 1 (*never true*) to 5 (*very often true*), plus three validity items. Consistent with the Dimensional Model of Adversity (23) and prior analytic strategies (44), we created two composite variables: Childhood Abuse: Sum of the Emotional Abuse and Physical Abuse subscale scores. Childhood Neglect: Sum of the Emotional Neglect and Physical Neglect subscale scores. Higher scores indicate greater severity of abuse or neglect. It is worth noting that, due to the fact that children who have suffered from sexual abuse are unlikely to voluntarily report such experiences (45, 46), and “many Chinese parents and teachers thought that questions about child sexual abuse were too sensitive and inappropriate for the adolescents to answe” (45), the dimension of sexual abuse was excluded in this study to increase the response rate and reduce reporting bias. In this sample, Cronbach’s alpha was.82 for the Abuse composite and.85 for the Neglect composite.

#### 2.2.2 Self-compassion (T1)

Self-compassion was measured using the Chinese adaptation of the Self-Compassion Scale (SCS) developed by Neff (47). The full scale consists of 26 items designed to tap six components: Self-Kindness, Self-Judgment, Common Humanity, Isolation, Mindfulness, and Over-Identification. Respondents rate each item (e.g., “I try to be understanding and patient towards those aspects of my personality I don’t like”) on a 5-point scale from 1 (*almost never*) to 5 (*almost always*). Following contemporary psychometric recommendations that argue against treating self-compassion as a unitary construct by simply reverse-scoring negative items (48, 49), we computed two separate composite scores: Positive Self-Compassion: The mean score of the three positive subscales: Self-Kindness, Common Humanity, and Mindfulness. Negative Self-Compassion: The mean score of the three negative subscales: Self-Judgment, Isolation, and Over-Identification. In the current sample, internal consistency was excellent for both composites (PSC: α = .91; NSC: α = .89).

#### 2.2.3 Non-suicidal self-injury (T2)

The frequency of NSSI was assessed using the Chinese version of the Inventory of Statements about Self-Injury (ISAS) (50, 51). For this study, we utilized Part I of the ISAS, which assesses the lifetime and recent frequency of 12 common NSSI behaviors (e.g., cutting, burning, hitting oneself, severe scratching). Items are ranked on a 4‐point Likert scale ranging from 1 (never) to 4 (six times or more) higher total scores indicating a higher frequency of NSSI. Cronbach’s alpha in this study was.94.

#### 2.2.4 Cyber aggression (T2)

Cyber aggression was measured using the revised Adolescent Cyber Aggression Scale (ACAS) (52). This 15-item scale was developed and validated in Chinese contexts to assess proactive aggression enacted through digital means. It comprises two correlated subscales measuring different forms of online aggression: Overt Cyber Aggression (7 items): e.g., “I insult and curse others when playing online games.” Relational Cyber Aggression (8 items): e.g., “I spread rumors about someone or an organization online.” Participants rate how often they have engaged in each behavior on a 4-point Likert scale from 1 (*never*) to 4 (*always*), referring to the past six months. In this sample, internal consistency was.87 for the Overt subscale and.94 for the Relational subscale.

### 2.3 Data analysis

Data preparation and preliminary analyses were conducted using SPSS Version 26.0. Primary hypothesis testing involving structural equation modeling (SEM) was performed using Mplus Version 8.3.

Descriptive statistics (means, standard deviations) and bivariate Pearson correlations among all primary study variables and demographic covariates (gender, age) were computed. We tested our hypotheses using path analysis within the SEM framework. All models used maximum likelihood (ML) estimation. To account for the longitudinal design, all paths were specified from T1 predictors to T2 outcomes. Gender and age were included as statistical controls on all endogenous variables.

## 3 Results

### 3.1 Preliminary analyses: descriptive statistics and correlations

Descriptive statistics (means and standard deviations) and bivariate Pearson correlations for all primary study variables are presented in **Table 1**. Age and gender were included as demographic covariates in subsequent models, and their correlations with the study variables are also reported.

**Table 1:** Descriptive statistics and bivariate correlations for study variables.

Variables	*M*	*SD*	1	2	3	4	5	6
1. T1 Childhood Abuse	12.94	4.25						
2. T1 Childhood Neglect	21.93	7.23	0.06^**^					
3. T1 Positive SC	19.49	5.50	0.01	-0.26^***^				
4. T1 Negative SC	16.27	5.30	0.18^***^	-0.06^**^	0.54^***^			
5. T2 NSSI	12.96	4.25	0.07^**^	0.09^***^	-0.06^**^	0.07^**^		
6. T2 Cyber Aggression	16.57	3.90	0.05^*^	0.09^***^	-0.05^**^	0.07^**^	0.32^***^	
Gender	1.53	0.50	-0.03	-1.14^***^	0.09^***^	-0.04	-0.08^***^	-1.58^***^
Age	18.94	1.94	-0.11^***^	0.15^***^	-0.09^***^	-0.12^***^	-0.02	0.02

T1 = Time 1; T2 = Time 2; SC = Self-Compassion; NSSI = Non-Suicidal Self-Injury. Gender: 1 = Male, 2 = Female. *p <.05. **p <.01. ***p <.001.

Regarding the primary variables, T1 Childhood Abuse was positively correlated with T1 NS(*r* = 0.18, *p* < 0.001), T2 NSSI(*r* = 0.07, *p* = 0.01), and T2 Cyber Aggression (*r* = 0.05, *p* = 0.03), but was not significantly correlated with T1 PSC (*r* = 0.01, *p* = 0.56). T1 Childhood Neglect was negatively correlated with both T1 PSC (*r* = -.26, *p* <.001) and T1 NSC (*r* = -.06, *p* = .01), and positively correlated with both T2 NSSI (*r* = .09, *p* <.001) and T2 Cyber Aggression (*r* = .09, *p* <.001). As expected, T1 PSC and T1 NSC were positively correlated (*r* = .54, *p* <.001), supporting their conceptual relatedness while affirming their distinctiveness. T1 PSC was negatively associated with both T2 NSSI (*r* = -.06, *p* = .01) and T2 Cyber Aggression (*r* = -.05, *p* = .01). Conversely, T1 NSC was positively associated with both T2 NSSI (*r* = .07, *p* = .01) and T2 Cyber Aggression (*r* = .07, *p* = .01). Finally, a strong positive correlation was observed between T2 NSSI and T2 Cyber Aggression (*r* = .32, *p* <.001).

### 3.2 Direct effects of childhood maltreatment on NSSI and cyber aggression

**Figure 1:**
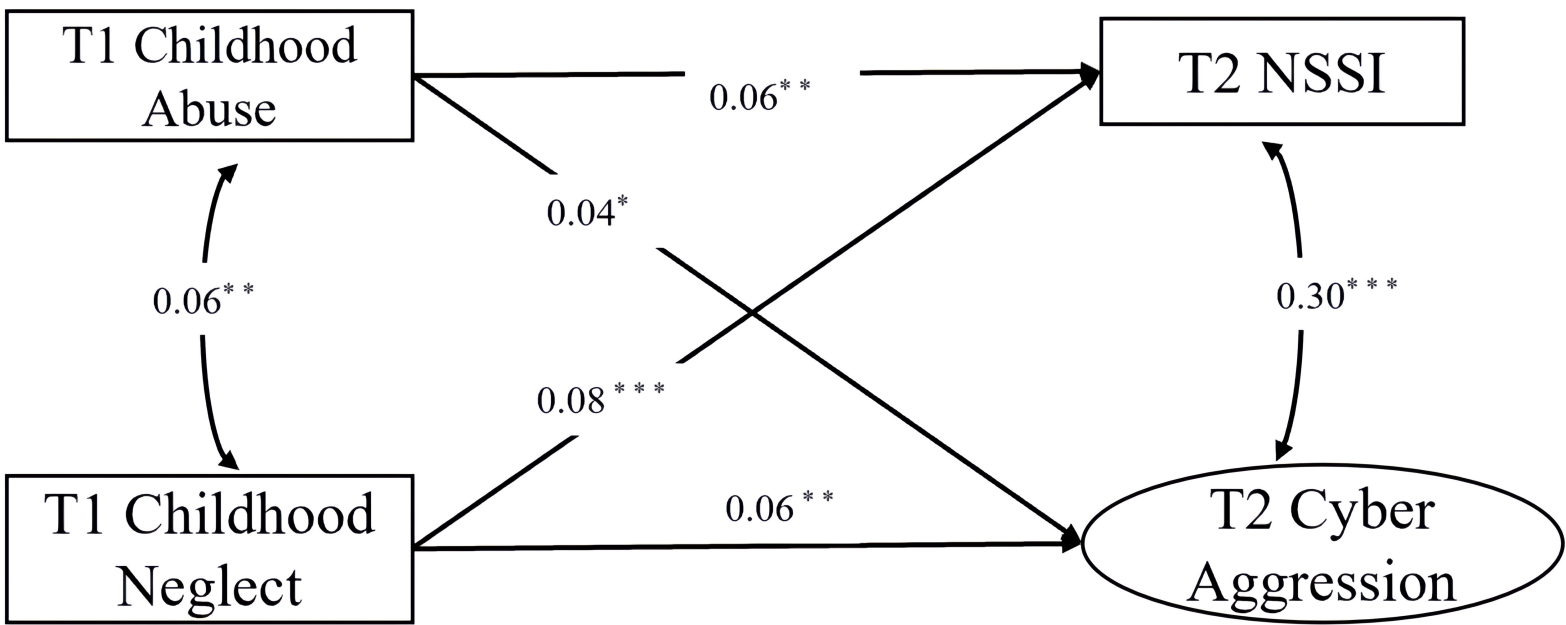
Direct effects model of childhood maltreatment on NSSI and cyber aggression. Standardized path coefficients are shown. T1 = Time 1; T2 = Time 2; NSSI = Non-Suicidal Self-Injury. *p <.05. **p <.01. ***p <.001.

Prior to testing the mediation model, a structural equation model (SEM) was constructed to examine the direct effects of T1 Childhood Abuse and T1 Childhood Neglect on T2 NSSI and T2 Cyber Aggression, controlling for gender and age (see **Figure 1**). The model also included correlations between the residuals of the two maltreatment predictors and between the residuals of the two outcome variables. The model demonstrated acceptable fit to the data: χ²(2) = 46.08, *p* <.001; CFI = .96; TLI = .92; RMSEA = .098 (90% CI:.086,.110). While the RMSEA value was slightly above the conventional.08 threshold, the CFI and TLI indicated good fit. It is recognized that RMSEA can be sensitive to model complexity and sample size, and other indices (CFI/TLI) are often prioritized in such contexts (53). Given the primary goal of testing theoretically specified paths rather than achieving perfect fit, and considering the good values on CFI and TLI, the model was deemed acceptable for interpretation.

The path coefficients revealed distinct patterns for abuse and neglect. T1 Childhood Abuse was a significant positive predictor of T2 Cyber Aggression (β = .04, *p* = .035) and T2 NSSI (β = .06, *p* = .006) when controlling for neglect and demographics; T1 Childhood Neglect was a significant positive predictor of both T2 NSSI (β = .08, *p* <.001) and T2 Cyber Aggression (β = .06, *p* = .007).

### 3.3 Mediating role of self-compassion

**Figure 2:**
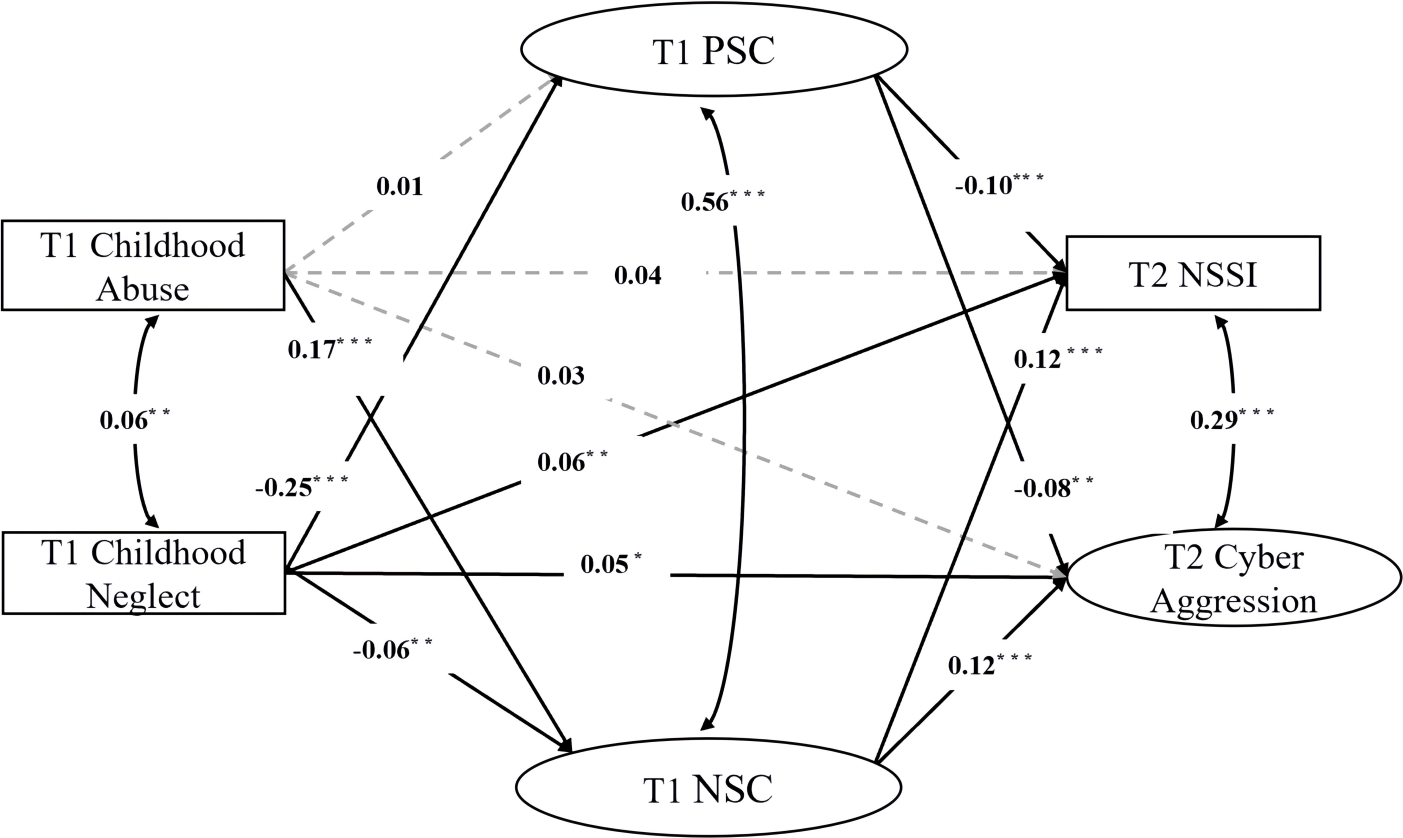
Structural equation model of self-compassion mediating the relationship between childhood maltreatment, NSSI, and cyber aggression. Standardized path coefficients are shown. T1 = Time 1; T2 = Time 2; PSC = Positive Self-Compassion; NSC = Negative Self-Compassion; NSSI = Non-Suicidal Self-Injury. For clarity, correlations between predictors/outcomes and covariate paths are not shown. * p <.05. ** p <.01. *** p <.001.

The full mediation model was tested next, incorporating T1 PSC and T1 NSC as mediators between the two forms of childhood maltreatment and the two behavioral outcomes (see **Figure 2**). Gender and age were included as covariates on all endogenous variables. The model demonstrated good fit to the data: χ²(34) = 639.88, *p* <.001; CFI = .97; TLI = .90; RMSEA = .088 (90% CI:.077,.099). The TLI was marginally below.90, but the CFI and RMSEA met acceptable-to-good fit criteria.

#### 3.3.1 Path coefficients in the mediation model

The standardized path coefficients from the SEM are presented in **Figure 2**. After accounting for the mediators and covariates, the direct paths from T1 Childhood Abuse to T2 NSSI (β = .04, *p* = .064) and T2 Cyber Aggression (β = .03, *p* = .230) became non-significant. The direct paths from T1 Childhood Neglect to both T2 NSSI (β = .06, *p* = .009) and T2 Cyber Aggression (β = .05, *p* = .043) remained significant.

Examining the paths to the mediators, T1 Childhood Abuse significantly and positively predicted T1 NSC (β = .17, *p* <.001) but did not significantly predict T1 PSC (β = .01, *p* = .476). Conversely, T1 Childhood Neglect significantly and negatively predicted both T1 PSC (β = -.25, *p* <.001) and T1 NSC (β = -.06, *p* = .004).

Regarding the paths from the mediators to the outcomes, T1 PSC significantly and negatively predicted both T2 NSSI (β = -.10, *p* <.001) and T2 Cyber Aggression (β = -.08, *p* = .002). T1 NSC significantly and positively predicted both T2 NSSI (β = .12, *p* <.001) and T2 Cyber Aggression (β = .12, *p* <.001).

#### 3.3.2 Significance of indirect effects

The significance of the specific indirect pathways was tested using bias-corrected bootstrapping with 5,000 resamples. The results are summarized in **Table 2**.

**Table 2:** Bootstrap analysis of indirect effects in the mediation model.

Indirect pathway	Standardized estimate	SE	95% Bootstrap CI	
			Lower	Upper
T1 Abuse → T1 PSC → T2 NSSI	-0.001	0.003	-0.007	0.003
T1 Abuse → T1 PSC → T2 Cyber Aggression	-0.001	0.002	-0.006	0.002
T1 Abuse → T1 NSC → T2 NSSI	0.019^***^	0.005	0.010	0.032
T1 Abuse → T1 NSC → T2 Cyber Aggression	0.020^***^	0.005	0.011	0.032
T1 Neglect → T1 PSC → T2 NSSI	0.025^***^	0.007	0.012	0.039
T1 Neglect → T1 PSC → T2 Cyber Aggression	0.020^**^	0.007	0.007	0.035
T1 Neglect → T1 NSC → T2 NSSI	-0.004^*^	0.002	-0.015	-0.001
T1 Neglect → T1 NSC → T2 Cyber Aggression	-0.007^*^	0.003	-0.015	-0.002

T1 = Time 1; T2 = Time 2; PSC = Positive Self-Compassion; NSC = Negative Self-Compassion; NSSI = Non-Suicidal Self-Injury; CI = Confidence Interval.

*p <.05. **p <.01. ***p <.001.

As hypothesized (H2a), T1 Childhood Abuse had a significant positive indirect effect on both T2 NSSI and T2 Cyber Aggression through T1 NSC (ab → NSC → NSSI: β = .019, 95% CI [.010,.032]; ab → NSC → Cyber Aggression: β = .020, 95% CI [.011,.032]). The indirect effects through T1 PSC were not significant.

Also as hypothesized (H2b), T1 Childhood Neglect had a significant positive indirect effect on both outcomes through T1 PSC (neglect → PSC → NSSI: β = .025, 95% CI [.012,.039]; neglect → PSC → Cyber Aggression: β = .020, 95% CI [.007,.035]). This indicates that higher neglect was associated with lower PSC, which in turn was associated with higher NSSI and cyber aggression.

However, an unexpected but significant finding emerged: T1 Childhood Neglect also had a significant *negative* indirect effect on both outcomes through T1 NSC (neglect → NSC → NSSI: β = -.004, 95% CI [-.015, -.001]; neglect → NSC → Cyber Aggression: β = -.007, 95% CI [-.015, -.002]). This counterintuitive result suggests that, in this model, higher neglect was associated with *lower* NSC, which then predicted *lower* levels of NSSI and cyber aggression. This suppressor effect is explored in the discussion.

## 4 Discussion

The present longitudinal study sought to advance the understanding of dual harm—the co-occurrence of self- and other-directed aggression—by investigating the distinct pathways from childhood maltreatment to NSSI and cyber aggression in a large sample of Chinese university students. Grounded in the Cognitive-Emotional Model of Dual Harm (14) and the Dimensional Model of Adversity (23), the study proposed self-compassion as a critical mediating mechanism. The results provide nuanced support for our hypotheses while revealing complexities in how different forms of early adversity shape later psychological and behavioral outcomes.

### 4.1 The roles of abuse and neglect in dual harm

Consistent with developmental psychopathology frameworks and the dual harm model (14), both childhood abuse and neglect emerged as significant distal predictors of later harmful behaviors, underscoring their shared role in elevating long-term risk. The direct effects model revealed that both forms of maltreatment independently contributed to the prediction of NSSI and cyber aggression six months later, even when controlling for each other. Specifically, childhood neglect was a robust predictor of both outcomes, a finding that aligns with literature emphasizing its link to broad deficits in emotion regulation, social cognition, and self-concept development due to the profound absence of necessary care and stimulation (54). Similarly, childhood abuse also demonstrated a significant direct association with both NSSI and cyber aggression. This pattern suggests that, at a broad behavioral level, both threat-based and deprivation-based adversities constitute potent risk factors for the subsequent emergence of dual harm.

This convergence in direct effects highlights a crucial clinical reality: a history of any major childhood maltreatment should alert professionals to a heightened vulnerability for a spectrum of dysregulated behaviors, encompassing both self-directed and other-directed harm. The experience of abuse, characterized by direct interpersonal violation and the modeling of aggression, may not only foster externalizing pathways (19) but also create a foundational distress that can manifest inwardly as NSSI. While the magnitude of the standardized coefficients for neglect was slightly larger than for abuse, the key finding is the statistical significance of both, rejecting a simple dichotomy where one type of maltreatment exclusively predicts one type of harm.

### 4.2 Self-compassion as a divergent mediating pathway

The most significant contribution of this study lies in unpacking the mediating role of self-compassion’s two dimensions. The results clearly demonstrate that abuse and neglect influence self-relating in meaningfully different ways, which subsequently channel risk toward dual harm.

#### 4.2.1 The pathway from childhood abuse: negative self-compassion

As hypothesized, childhood abuse was a significant predictor of higher levels of negative self-compassion (self-judgment, isolation, over-identification), which in turn predicted greater engagement in both NSSI and cyber aggression. This pathway illustrates the cognitive-emotional mechanism proposed by Shafti et al. (14). Abusive experiences, where harm is actively inflicted, likely foster an internal working model of the self as deserving of criticism and contempt (39). The child internalizes the abuser’s voice, leading to a pervasive pattern of self-condemnation and a ruminative focus on personal flaws and distress (15). This negative self-compassionate stance is emotionally toxic. It amplifies suffering, inhibits self-soothing, and creates a state of high inner tension.

To escape this aversive state, individuals may resort to NSSI as a desperate form of self-punishment or emotional regulation (11). Simultaneously, the anger and frustration born from this harsh self-scrutiny and perceived injustice may be projected outward as cyber aggression, serving as a displaced expression of rage or a maladaptive attempt to regain a sense of power and control (35). This internalization of a persecutory inner critic following threat-based adversity is a core tenet of psychodynamic and cognitive models of trauma, further validating the cross-cultural relevance of this pathway.

#### 4.2.2 The pathway from childhood neglect: positive self-compassion

Also as hypothesized, childhood neglect exerted a significant indirect effect on dual harm by eroding positive self-compassion. When caregivers are emotionally absent or fail to meet basic physical needs, the child does not receive the nurturing necessary to develop an internalized capacity for self-care and kindness (55). The absence of comfort leads to an absence of learning how to self-comfort. Consequently, these individuals enter adulthood with a deficit in the fundamental skill of treating themselves with warmth and understanding during times of difficulty. This lack of positive self-compassion leaves them vulnerable; when faced with stress, failure, or emotional pain, they have no compassionate inner refuge to turn to. The resulting emotional overwhelm and perceived lack of coping resources can then manifest as NSSI, a stark alternative to self-kindness, and as cyber aggression, potentially driven by unmet needs for connection or poor emotion regulation stemming from this self-care deficit.

#### 4.2.3 The complex role of negative self-compassion in neglect

An unexpected and intriguing finding was the significant negative indirect path from neglect to dual harm through negative self-compassion. While the zero-order correlation between neglect and negative self-compassion was negative but weak, in the structural model accounting for shared variance with abuse and positive self-compassion, this path indicated that higher neglect was associated with lower negative self-compassion, which then predicted lower NSSI and cyber aggression. This counterintuitive result merits careful interpretation. It may point to a phenomenon where severe emotional and physical deprivation leads not to an active, critical inner voice, but to a kind of emotional numbing, emptiness, or a lack of developed self-awareness altogether (56). Unlike abuse, which imposes a hostile internal dialogue, neglect may represent a void where such a dialogue—even a negative one—fails to fully form. In other words, neglected individuals might not engage in high levels of self-judgment or ruminative over-identification simply because the capacity for complex, sustained self-reflection (including negative reflection) has been stunted. This “protective” effect against negative self-compassion, however, should not be mistaken for health; it coexists with a profound lack of positive self-compassion and directly predicts harm through that primary pathway. This finding underscores the critical importance of separately modeling the two dimensions of self-compassion and cautions against viewing low negative self-compassion in isolation as an adaptive trait.

### 4.3 Theoretical implications and integration

These differential mediation pathways offer strong, nuanced support for the Dimensional Model of Adversity (23). The model posits that threat (abuse) and deprivation (neglect) impact different neurodevelopmental and learning systems. Our findings extend this to the domain of socio-emotional learning and self-schema development. Threat appears to actively shape a specific, maladaptive form of self-relating (high negative self-compassion), akin to learning a hyper-vigilant and self-critical stance. Deprivation, conversely, seems to prevent the development of an adaptive form of self-relating (low positive self-compassion), akin to a failure to learn self-soothing. Both outcomes increase the risk for psychopathology, but they do so via distinct psychological channels, which our study maps onto specific behavioral outcomes (dual harm).

Furthermore, the results validate and elaborate the Cognitive-Emotional Model of Dual Harm. We provide empirical evidence that early adversity shapes core cognitive-emotional schemas (self-compassion), which subsequently govern responses to distress. The model’s emphasis on shared vulnerability factors for self- and other-directed harm is supported by the fact that both positive and negative self-compassion predicted both NSSI and cyber aggression. Whether the schema deficit is the absence of kindness or the presence of cruelty, the behavioral consequence can be harm directed inward, outward, or both.

### 4.4 Practical implications

The clinical and preventive implications are significant. First, routine screening for childhood maltreatment in university mental health services is warranted, with attention to distinguishing between experiences of abuse and neglect. Second, interventions must be tailored. For students with histories of abuse, therapy should directly target the ingrained inner critic and the patterns of negative self-compassion. Modalities like Compassion-Focused Therapy (CFT), developed in Western clinical contexts, are explicitly designed to de-shame and cultivate self-kindness in the face of high self-criticism and have shown efficacy in treating trauma-related disorders (57). For those with histories of neglect, the therapeutic goal may be more foundational: to foster the very capacity for self-care, warmth, and mindful self-awareness that was never instilled. Building positive self-compassion skills from the ground up would be a primary focus.

More broadly, university well-being programs can integrate psychoeducation and skills training in self-compassion as a universal preventive measure. Teaching students how to relate to themselves with kindness during academic setbacks, social difficulties, and emotional pain could serve as a buffer, potentially reducing the incidence of both self-harm and interpersonal cyber aggression.

### 4.5 Limitations and future directions

Several limitations must be acknowledged. First, all data were self-reported, which may introduce biases related to memory, social desirability, or current mood. Retrospective reports of childhood maltreatment can be influenced by recall bias and current psychological state. Similarly, self-reports of sensitive behaviors like NSSI and cyber aggression may be under-reported due to stigma. Future studies would benefit from multi-method, multi-informant assessments, including clinical interviews, other-report measures, or official records where feasible, to mitigate these biases. Second, while longitudinal, the study spanned only six months. Longer-term follow-ups are needed to examine the stability of these pathways across broader swaths of emerging adulthood. A related and important limitation is the measurement of the proposed mediator (self-compassion) at the same time point (T1) as the predictor (maltreatment). Although our design establishes that T1 maltreatment predicts T2 outcomes, the concurrent assessment of the mediator prevents us from conclusively demonstrating that changes in self-compassion temporally follow maltreatment and precede the outcomes. A stronger design would involve at least three waves, measuring maltreatment, then mediators, then outcomes separately over time to more rigorously test the proposed mediation sequence. Third, we excluded sexual abuse from our assessment due to cultural sensitivities. Although methodologically justified for this context, it means our construct of “abuse” is incomplete, and future research in contexts where it is feasible should aim to include it. Fourth, we controlled only for age and gender. Other potentially important covariates, such as family socioeconomic status, prior psychiatric history, or concurrent symptoms of depression and anxiety, were not measured. Their absence means we cannot rule out the possibility that part of the observed relationships are confounded by these unmeasured variables. Future studies should incorporate a broader set of demographic and clinical covariates to isolate the unique contributions of maltreatment types and self-compassion. Finally, although our primary SEM models showed acceptable fit based on CFI and TLI indices, the RMSEA values were modest. This suggests room for improvement in the specified relationships.

Future research should build upon these findings in several key directions. First, to address causality and temporal dynamics, micro-longitudinal designs such as Ecological Momentary Assessment could be used to examine how daily fluctuations in state self-compassion mediate the link between momentary stressors and urges for both NSSI and online aggression among individuals with maltreatment histories. Second, the differential pathways we identified call for tailored intervention research. Randomized Controlled Trials are needed to test whether interventions specifically targeting negative self-compassion are more effective for abuse survivors, while interventions focused on building foundational positive self-compassion skills are more effective for neglect survivors, in reducing dual harm outcomes. Third, longer-term multi-wave studies from childhood into adulthood are essential to validate the developmental sequencing of these pathways. Finally, cross-cultural comparative studies are warranted to examine whether the strength of these mediated pathways varies across cultural contexts with different norms regarding self-criticism, emotional expression, and parenting.

### 4.6 Conclusion

In conclusion, this study moves beyond a monolithic view of childhood maltreatment to demonstrate that abuse and neglect confer risk for dual harm through emotionally distinct tributaries. Abuse fuels the fire of negative self-compassion—a critical, isolating inner world that propels individuals toward hurting themselves and others. Neglect, by contrast, starves the development of positive self-compassion—leaving individuals without an inner sanctuary of kindness, thereby also increasing vulnerability to harm. Both paths converge on the same river of behavioral dysregulation. By identifying these differential pathways, we not only enrich developmental theories of adversity but also light the way for more precise, compassionate, and effective interventions aimed at healing the wounds of childhood and preventing their tragic recurrence in the lives of young adults.

## Data Availability

The raw data supporting the conclusions of this article will be made available by the authors, without undue reservation.
